# Supplementary material for: Towards development of a statistical framework to evaluate myotonic dystrophy type 1 mRNA biomarkers in the context of a clinical trial
Source: PLoS One. 2020 Apr 14;15(4):e0231000. doi: 10.1371/journal.pone.0231000 (PMC7156058; doi:10.1371/journal.pone.0231000)
Supplement: S1 Appendix — (PDF) [file pone.0231000.s001.pdf]

S1 Appendix. Code & how to run it. All of the computer programs written for this study can be found on github.

The following instructions should allow one to independently verify results of our simulations (also available in the repository).

We advise that all of this code be run on a machine with 64 GB of RAM or more, given that some parts of the pipeline can use up in excess of 32 GB of RAM. We were able to successfully execute the entire analysis using an AWS "m5.4xlarge" EC2 instance. We found the default amount of storage, 8 GB, to be insufficient to store both the primary data and intermediate computations. We increased the amount of storage to 100 GB. We remove all networking restrictions on the instance, to allow for remote access of jupyter notebooks, which contain our pipeline.

We had to apply the following shell commands to set up the machine:

1. sudo apt-get update
2. sudo apt-get upgrade
3. sudo apt-get install python3-pip
4. git clone https://github.com/picrin/clinical_applications.git
5. pip3 install jupyter

All data and metadata used in this study are available in publicly accessible s3 bucket, with the following paths: "dm1-biomarkers/CEL", "dm1-biomarkers/annotations" respectively. These datasets need to be copied to the root of the "clinical_applications" repository as directories "CEL" and "annotations" respectively.

All required third-party dependencies can be installed from the provided "requirements.txt"

Finally, "jupyter notebook −−ip 0.0.0.0 −−port 8888" can be issued to start the notebook server, which we can access remotely, using a DNS entry allocated for our EC2 instance and provided that communications on our chosen port is configured to be accessible through the AWS firewall.

We now run notebooks in the following order:

1. "01_parse_chip_data.ipynb". This part of the pipeline is responsible for determining probeset ids, sequences of probes and probe coordinates on the chip. It produces an intermediate file with data adhering to the following schema: "probeset, x, y, sequence".
2. "02_parse_csv_annotations.ipynb". Here, we determine "genomic" metadata, i.e. chromosomomal coordinates and strandedness.
3. "03_unpack_CEL_files.ipynb". Here we use our own contribution to Biopython to parse the binary CEL v4 file format, which is what all our microarray data uses.
4. "04_quantile_normalise.ipynb". Here we perform quantile normalisation of our microarray data.

5. "05_reannotate_probeset_level.ipynb". Here we verify Affymetrix's annotation. We determine that over 1% probes are incorrectly annotated. We discard these probes. We limit our attention to probes, which belong to chromosomes chr1-chr22, X, Y and the mitochondrial DNA (M).

6. "06_intervaltrees.ipynb". Here we carry out an exclusive filtering, choosing probes, which are identified by "gencode.v26lift37.annotation.gtf" as having "transcript_type" equal to "protein_coding", or "gene_type" equal to "protein_coding", as well as the exon type equal to "CDS" (Coding sequence) or "UTR" (5' or 3' untranslated regions).

7. "07_merge_annotation_experiment.ipynb". Here we produce a single file per human gene, as identified in GENCODE v26, with data from all participants for all probes for that gene.

8. "08_predictions.ipynb". Here we run our PLSR model to predict MAL from the microarray data.

9. "10_power_analysis.ipynb". Here we run power analysis presented in Results & Discussion.

10. "13_combine_p_values.ipynb". Here we combine p-values for a probeset of interest using a previously published p-value and one obtained in this study.
