# Supplementary material for: Towards development of a statistical framework to evaluate myotonic dystrophy type 1 mRNA biomarkers in the context of a clinical trial
Source: PLoS One. 2020 Apr 14;15(4):e0231000. doi: 10.1371/journal.pone.0231000 (PMC7156058; doi:10.1371/journal.pone.0231000)
Supplement: S2 Appendix — (PDF) [file pone.0231000.s002.pdf]

S2 Appendix. DM1-AS

ABLIM2, ALPK3, ANK2, ARFGAP2, ATP2A1, ATP2A2, BIN1, CACNA1S, CAMK2B, CAPN3, CAPZB, CLCN1, COPZ2, DMD, DTNA, FHOD1, GFPT1, IMPDH2, INSR, KIF13A, LDB3, MBNL1, MBNL2, MLF1, NFIX, NRAP, OPA1, PDLIM3, PHKA1, RYR1, SOS1, TBC1D15, TTN, TXNL4A, UBE2D3, USP25, VEGFA, VPS39
