# Supplementary material for: Towards development of a statistical framework to evaluate myotonic dystrophy type 1 mRNA biomarkers in the context of a clinical trial
Source: PLoS One. 2020 Apr 14;15(4):e0231000. doi: 10.1371/journal.pone.0231000 (PMC7156058; doi:10.1371/journal.pone.0231000)
Supplement: S3 Appendix — (PDF) [file pone.0231000.s003.pdf]

S3 Appendix. DM1-APA

ABCA1, AGL, ALG3, AMHR2, AP1G1, ARHGEF7, ASPH, ATP5E, BRSK2, BRWD1, CACNA1G, CACNB1, CDC42, CEBPA, CELF1, CHRNA1, CIRBP, CLDND1, COPS4, DAPK2, DES, DNAJB6, DST, DVL3, EZR, FASTK, GPS1, HDAC11, IDH3A, ILF3, KCNK7, KDELR1, KIF1B, KRBA1, LAMP2, LDB3, LMNA, MBNL2, MDN1, MEF2B, MEF2C, MEF2D, MGP, MORC3, MTCH1, MYH6, NDUFB10, NR2F1, NUP43, OSBPL1A, PCBD2, PCM1, PCMT1, PDLIM2, PDLIM5, PEBP4, PFKFB2, PIK3C2B, PLIN2, RAB24, RIN1, RTN2, SAMD4A, SETD3, SLC25A36, SMIM3, SNX1, SPATS2L, SPEG, SPTB, TBL2, TGFBI, TJP2, TMEM38B, TNNI1, TPM1, TPM2, TPM3, TTYH3, U2SURP
