# Supplementary material for: Towards development of a statistical framework to evaluate myotonic dystrophy type 1 mRNA biomarkers in the context of a clinical trial
Source: PLoS One. 2020 Apr 14;15(4):e0231000. doi: 10.1371/journal.pone.0231000 (PMC7156058; doi:10.1371/journal.pone.0231000)
Supplement: Attachment — Submitted filename: Response_to_reviewers_2_Kurkiewicz_DMBDI.pdf [file pone.0231000.s005.pdf]

Response to reviewers

Towards development of a statistical framework to evaluate myotonic dystrophy type 1 mRNA biomarkers in the context of a clinical trial

We'd like to thank the academic editor Ruben Artero for providing useful advice on our submission, coordinating the revision process and soliciting insightful comments from the reviewers.

We'd like to thank Juan Antonio Carbonell-Asíns and the anonymous reviewer for sharing a critical perspective on our work.

Editorial and reviewer comments are highlighted in moss green.

Whenever the manuscript text is cited, changes made are highlighted in electric yellow.

Response to further comments by Juan Antonio Carbonell-Asíns

Reviewer #2: I would like to thank the authors for all clarifications in the reviewing of the manuscript entitled “Towards development of a statistical framework to evaluate myotonic dystrophy type 1 mRNA biomarkers in the context of a clinical trial” by Kurkiewicz et al. However I have still some follow-up comments regarding two of my previous remarks.

1. R2 is used to evaluate predictive power of each model but this strategy does not penalize by number of predictors. Other methods are suggested as, for instance, AIC/BIC.

I can now understand the aim of using R2 but still wonder if the relation between predicted and observed is linear. Moreover, beta coefficient for the slope should be equal to one and beta coefficient for intercept equal to 0. I suggest relating only to root-mean-square deviation (RMSD) and its confidence interval for model comparison.

References to summary statistics and outcomes of statistical tests, such as R2 and p-value made in the "Performance of PLSR-based MAL predictors in muscle and blood" subsection of the "Results & Discussion" section of this submission were in relation to a total of 8 computationally intensive simulations, each consisting of 10000 split-train-predict cycles. Due to implementation and operational details (the simulation outcomes were written to a temporary file, with only summary statistics persisted long-term) we had to re-run these simulations to obtain RMSD measures. The simulations contain a randomised Cross Validation (CV) step, which means that re-running the simulations results in a different set of predictions, and a slightly different value of the summary statistics (e.g. R2, RMSD). Due to a large number of repetitions present in the simulation (10000), both estimates obtained in the previous and the current run gave comparable estimates of the summary statistics and corresponding statistical tests.

With the new set of simulations present, we could address the improvements suggested by the reviewer. Specifically, we computed the RMSD statistic for all experiments. These are now displayed alongside R2 in the results section of the manuscript. Reassuringly, RMSD results are consistent with R2, i.e. whenever R2 shows stronger correlations (higher values closer to

1), RMSD shows lower error (lower values closer to 0). The only exception to this rule occurs when considering TNNI1 as a biomarker measured in blood, which gives us an unusually high value of RMSD. We decided that due to this very good agreement of RMSD with R2, we will keep the discussion centered around R2.

We could not obtain the confidence intervals analytically for any of the computed statistics, and our attempt to proceed using bootstrap proved difficult due to the limitations of available packages. Given the design of this study, the major concern with regards to the confidence intervals for the obtained estimates of the summary statistics is that 10000 repetitions of the simulation could not have been sufficient to achieve satisfactory convergence. To reassure ourselves and the reviewer that this is not the case we repeated one of the experiments 9 times, and recorded deviation of the obtained R2 in each repetition of the experiment from the combined measure across all experiments. When expressed as percentage deviation, most of the individual estimates of the statistic were within 1.5% of the combined estimate: `[-3.000908592652183, -1.7353343619689265, -0.9303304379017678, 0.2308480928837935, 0.28157327896959794, 0.8483091370570505, 1.0581875095901017, 1.2051289892728774, 2.0425263847494874]`. We consider this amount of error in the estimate to be negligible, and decided to proceed without computing confidence intervals.

2. Splitting data with only 28/35 patients may cause bias in estimations. (Xu, Y., & Goodacre, R., 2018). So, I would like to recommend using all patients and conduct a standard model diagnostic. No model diagnosis has been provided.

I understand the difficulty of increasing the sample size but statistically speaking this is an important limitation of the study as PLS suffers from reduced accuracy with small dataset which is furtherly aggravated when partitioning the dataset. My major concern is the justification that the sample size to evaluate myotonic dystrophy type 1 mRNA biomarkers is big enough.

We agree with the reviewer that a sample size larger than 27 individuals would have been desirable. In fact, the original design of the study planned a collection of blood and tissue samples from the total of 100 individuals, but due to difficulties with participant enrolment the total number of samples collected was only 28 muscle biopsies and 35 blood samples from 36 individuals for the total of 27 complete (blood + tissue) samples.

In fact, obtaining a good sample size for an invasive myotonic dystrophy type 1 research study is often difficult due to the rarity of the disease. Even well-funded clinical trials may struggle with patient enrolment. For example, a recent clinical trial of an antisense oligonucleotide for DM1 used 8 clinical centres for the enrolment of only 29 patients: https://www.myotonic.org/sites/default/files/pages/files/Laurence-Mignon_IONIS-Update_2018-Conference.pdf

Simulation confirms the intuition that a better model could be obtained by simply training on a larger dataset. Specifically, R2 of 28.5% was reported for a model which used 18/9 as the training/testing split for the 27 individuals. Using the same model with 14/13 as a splitting strategy, performance of the model measured R2 to be only 17.6%.

We now discuss these considerations in the Limitations section:

Small sample size. In the study we obtained a complete tissue collection from only 27 individuals. Intuitively, this is a small sample size when judged by a typical size of a phase 1 clinical trial. Simulation confirms the intuition that a better model could be obtained by simply training on a larger dataset. Specifically, R2 of 28.5% was reported for a model which used 18/9 as the training/testing split for the 27 individuals. Using the same model with 14/13 as a splitting strategy, performance of the model measured R2 to be only 17.6%. We would like to note that our estimate is most likely conservative, and a larger sample size would likely give us a higher predictive strength.
