# Supplementary material for: Towards development of a statistical framework to evaluate myotonic dystrophy type 1 mRNA biomarkers in the context of a clinical trial
Source: PLoS One. 2020 Apr 14;15(4):e0231000. doi: 10.1371/journal.pone.0231000 (PMC7156058; doi:10.1371/journal.pone.0231000)
Supplement: Attachment — Submitted filename: Response to reviewers Kurkiewicz DMBDI.docx [file pone.0231000.s004.docx]

Response to reviewers

Towards development of a statistical framework to evaluate myotonic dystrophy type 1 mRNA biomarkers in the context of a clinical trial

We'd like to thank the academic editor Ruben Artero for providing useful advice on our submission, coordinating the revision process and soliciting insightful comments from the reviewers.

We'd like to thank Juan Antonio Carbonell-Asíns and the anonymous reviewer for sharing a critical perspective on our work.

Editorial and reviewer comments are highlighted in moss green.

Whenever the manuscript text is cited, changes made are highlighted in electric yellow.

# Response to the comments by the academic editor

1. When submitting your revision, we need you to address these additional requirements.

Please ensure that your manuscript meets PLOS ONE's style requirements, including those for file naming. The PLOS ONE style templates can be found at

http://www.journals.plos.org/plosone/s/file?id=wjVg/PLOSOne_formatting_sample_main_body.pdf and http://www.journals.plos.org/plosone/s/file?id=ba62/PLOSOne_formatting_sample_title_authors_affiliations.pdf

We've renamed the files to comply with the style requirements of PLOS ONE. We decided to automatically accomplish compliance with many aspects of the style requirements by using the recommended latex template for a PLOS ONE submission:

<https://journals.plos.org/plosone/s/latex#loc-plos-template>

2. Thank you for including your ethics statement:

"Collection, data analysis and public data release of human samples were approved by Institutional Review Boards of clinicians who contributed to this study, as arranged with the Marigold Foundation. The contributing clinicians were: Jack Puymirat, Benedikt Schoser, Tetsuo Ashizawa and Charles Thornton. Anonymised data from the study has been in the public domain since 31st of May 2019: https://www.ebi.ac.uk/arrayexpress/experiments/E-MTAB-7983/"

a. Please amend your current ethics statement to include the full name of the ethics committee/institutional review board(s) that approved your specific study.

b. Once you have amended this/these statement(s) in the Methods section of the manuscript, please add the same text to the “Ethics Statement” field of the submission form (via “Edit Submission”).

For additional information about PLOS ONE ethical requirements for human subjects research, please refer to http://journals.plos.org/plosone/s/submission-guidelines#loc-human-subjects-research

We've amended our ethics statement to include the full name of all ethical committees who approved our study. We've included this statement as a paragraph in Materials and Methods section of our submission:

The study was approved by the ethics committee of the Faculty of Medicine at LMU Munich, Germany; University of Rochester Research Subjects Review Board, Rochester, New York and University of Florida Institutional Review Board, Gainesville, Florida, USA.

3. Thank you for stating the following in the Competing Interests section:

"Adam Kurkiewicz

Declares ownership of Illumina and PacBio shares.

Anneli Cooper

Has served on a Scientific Advisory Board for AstraZeneca (Trypanosomiasis).

Sarah Cumming

None declared

Berit Adam

None declared

Ralf Krahe

None declared.

Jack Puymirat

None declared

Benedikt Schoser

Benedikt Schoser is member of the Neuromuscular advisory board of Audentes Therapeutics, USA, and Scientific advisory of Nexien BioPharm, USA. He received speaker honoraria from Sanofi Genzyme, Amicus Therapeutics, Lupin Pharmaceuticals, and Kedrion. He received an unrestricted research grant from Sanofi Genzyme USA (2016-2019), Greenovation FRG (2017-2020), and from the Marigold foundation (2014)

Lubov Timchenko

None declared.

Tetsuo Ashizawa

1. My wife has stocks and stock options of BIOPATH Holdings, Inc.

2. One US and international patent approved.

3. Grants from NIH, the Myotonic Dystrophy Foundation, the National Ataxia Foundation and Biogen.

4. I am a member of the advisory board for the National Ataxia Foundation and that for the Myotonic Dystrophy Foundation.

Simon Rogers

None declared

John McClure

None declared

Darren G Monckton

Professor Monckton has been a scientific consultant and/or received an honoraria or stock options from Biogen Idec, AMO Pharma, Charles River, Vertex Pharmaceuticals, Triplet Therapeutics, LoQus23, BridgeBio, Small Molecule RNA and Lion Therapeutics. Professor Monckton also had a research contract with AMO Pharma and CHDI has received research grants from the European Union, European Huntington Disease Network, National Institute of Health, Muscular Dystrophy UK and the Myotonic Dystrophy Support Group.

Professor Monckton is on the Scientific Advisory Board of the Myotonic Dystrophy Foundation, is a scientific advisor to the Myotonic Dystrophy Support Group and is a vice president of Muscular Dystrophy UK.

Charles Thornton

Dr. Thornton has received sponsored research support from Ionis Pharmaceuticals, Biogen, Genzyme, and Dyne Therapeutics, and research grants from the National Institutes of Health, Food and Drug Administration, Muscular Dystrophy Association, and Myotonic Dystrophy Foundation. Dr Thornton has served on the Scientific Advisory Board for Dyne Therapeutics. "

Please confirm that this does not alter your adherence to all PLOS ONE policies on sharing data and materials, by including the following statement: "This does not alter our adherence to PLOS ONE policies on sharing data and materials.” (as detailed online in our guide for authors http://journals.plos.org/plosone/s/competing-interests). If there are restrictions on sharing of data and/or materials, please state these. Please note that we cannot proceed with consideration of your article until this information has been declared.

Please include your updated Competing Interests statement in your cover letter; we will change the online submission form on your behalf.

We've modified our cover letter to include the required statement.

Please note while forming your response, if your article is accepted, you may have the opportunity to make the peer review history publicly available. The record will include editor decision letters (with reviews) and your responses to reviewer comments.

We would like to make the peer review history publicly available.

While revising your submission, please upload your figure files to the Preflight Analysis and Conversion Engine (PACE) digital diagnostic tool, https://pacev2.apexcovantage.com/. PACE helps ensure that figures meet PLOS requirements.

We've revised our figures to achieve compliance with PACE.

# Response to the comments by the anonymous reviewer

1. The discussion of “effective repeat length” on page 5 could use better framing. The authors are referring to the fact that there is a relationship between repeat length and degree of splicing changes, and that in fact the most important therapeutic read out is return of correct AS vs reduction of the CTG repeat length. This should be more explicitly stated.

We have re-worded the paragraph on page 5, which now is:

It is well established that the primary determinant of both age at onset and many progressive DM1 symptoms is the CTG repeat length (Morales et al. 2012; Overend et al. 2019; Cumming et al. 2019). Separate efforts have shown that case/control status in DM1 results in detectable AS changes in the mRNA profile of skeletal muscle (Nakamori et al. 2013; Batra et al. 2014). Our work acts as a bridge unifying both lines of investigation. In this research, rather than regarding DM1 status as a binary variable, we view DM1 as a spectrum of disease, the severity of which is quantified by the length of the DM1 CTG repeat in any individual patient. Based on the knowledge of the causative effect of CTG expansion on downstream pathology, and the important role of AS defects in this pathology, we thought it should be possible to capture the effect, which the length of the CTG repeat has on mRNA expression in muscle into a simple statistical model, based on partial least squares regression (PLSR). Using the model we can predict the size of the DM1 CTG repeat from the mRNA profile significantly better than a random predictor. We propose that the model can serve as a valuable tool in evaluating efficacy of any treatment for DM1 as such treatment enters pre-clinical or clinical trials, by enabling investigators to directly quantify the treatment effect as measured by effective reduction of DM1 CTG repeat length rather than simply assessing the shift in AS of any one transcript. As disease severity is directly tied to CTG length, using this approach has the potential to yield more clinically meaningful interpretations of AS changes. For instance, an AS event that is dramatically shifted to the opposite extreme by even a small increase in the number of CTG repeats will yield a large signal on a case-control basis, but may not be closely tied to disease severity within the DM1 population and thus may not make a good reporter of an intervention as it may require an unrealistically large effect size to revert it to the non-disease associated range. However, as such AS event yields little discriminatory power with regard to CTG repeat within the DM1 population, it would not be selected as a major contributor to our predictive model. Thus, our model has the potential to enrich for AS events that have more direct clinical relevance rather than absolute changes.

2. More recent work has used RNA-seq to identify splicing changes (such as Wagner et al.). Can the authors provide a rationale for reverting to the microarray approach?

Our views align with the consensus in the field that RNA-seq is a more suitable approach to identify transcript-level changes in DM1. We consider the work of Wagner et al. to be of great importance. It was the first study to directly link concentrations of MBNL1 with specific splicing changes, using an ingenious approach involving tetracycline-inducible HA-MBNL1 expression system. The in vitro findings were confirmed with data obtained from muscle biopsies in a cohort, which included 44 DM1 patients.

The dataset we worked with was gathered as part of a large international collaboration, which began work in 2010, before RNA-seq protocols became widely available. The work took a long time to accomplish due to organisational hurdles and funding difficulties. Yet the study provides value and new information that previously wasn't available:

1. A distinctive feature of the study is repeat sizing of each individual. This allowed us to use the repeat length as a means to stratify study participants.
2. The study was performed on quadriceps, as opposed to the more common choice of the more severely affected TA. It is interesting to see that splicing changes can be detected to a sufficient degree even in this less severely affected muscle.
3. Confirming the existence of splicing methods using microarrays, an alternative, if less popular, technique can be seen as an added value of the study.

It is important to stress that the statistical framework proposed is not limited to microarrays only. The framework could equally well be applied to new data sets, including RNA-seq, as long as they feature a sized CTG repeat.

We have now added a short expansion in Results and Discussion:

We would also like to note that the statistical framework proposed here is not limited to the experimental technique used. The framework could equally well be applied to new data sets, including RNA-seq, as long as they feature a sized CTG repeat for each participant in the dataset.

3. It is not clear from the informatic approach what steps the authors took to correct for multiple comparisons? If not, this should be provided as a limitation.

Identification of genes with disrupted alternatively splicing was not the primary focus of the study. None of the claims in the study depends on gene-level statistical tests (p-values). The only place in the study where these tests are performed is the visualisation tool, available at <https://dmbdi.adamkurkiewicz.com> (see the tool in use here: <https://youtu.be/gvs5XtdYwE0>), where multiple comparison correction is performed across all probes in the gene (a similar correction could be easily implemented by the user of the tool across all genes in the study or genome-wide).

Multiple comparison correction could be performed at the level of tests checking statistical significance outputs of the PLSR model (page 12 and 13, Table 1 and 2). Naively, this would give a number of multiple comparisons to be 8 (4 p-values in Table 1, and 4 p-values in Table 2). This would, however, be quite non-standard and suffer from its own validity issues, in particular with regards to choosing the p-values that should be included in the comparison (e.g. should one include the outcome of TNNI1 predictor chosen post-hoc? Should blood p-values be included in this multiple comparisons?).

Overall we felt that reporting exact, uncorrected p-values up to 5 significant digits was the most meaningful way to communicate statistical significance of obtained results. We believe that the reader will be able to reason about uncorrected p-values more intuitively than with multiplicity corrected p-values, especially if they were computed with potentially controversial multiplicity constant. An added value of operating with uncorrected p-values is the wealth of theoretical results that can be applied when reasoning about them, e.g. uniform distribution of p-values under the null or Fisher's method of combining p-values. Please see a particular example of this type of reasoning on page 16 in the paragraph beginning "Finally, and returning back to statistics, we can ask (...)".

4. In the results, the authors do not comment on the strength of the correlation. Even though they are statistically significant, correlations <0.4 are modest at best. Please provide a qualitative rating of the correlations.

We have now expanded the Results section with a subsection "Qualitative characterisation of the strength of observed correlations":

The strength of observed correlations of CTG repeat length and effective CTG length as measured by AS defects needs to be put in the context of similar correlations observed in other studies. We should distinguish here studies looking at large cohorts with a wide variety of clinical symptoms, *e.g.* studies including both congenital and late-onset participants, from studies looking at a relatively narrow range of participants, *e.g*. adult-onset, ambulatory participants only. In the former, highly variable cohorts, CTG repeat length has been shown to explain a large fraction of the variance of such DM1 symptoms as age-at-onset (*e.g*., r^2^=0.640 in Morales *et al*.). It has been much less successful in the latter type of studies, where study selection criteria indirectly limit the range of observed CTG repeats.

The study described here is of the latter type, with selection criteria being adult-onset, ambulatory DM1 patients. In this context, and taking into account the modest size of this study, the most significant correlation, *i.e.* between effective CTG length as predicted from DM1-APA and the true CTG length as measured using PCR (R^2^=0.29) actually compares favourably with symptoms most strongly correlated in similar studies, such as correlation of CTG repeat length and grip strength (R^2^=0.443), pinch strength (R^2^=0.419) and ankle dorsiflexor strength (R^2^=0.202) (Overend *et al.*).

A tempting question to ask is whether the study presented here allows us to draw conclusions with regards to explainability of AS defects with CTG length. Unfortunately, the study design did not include any biological or technical replicates. As a result, we were unable to quantify the relative contributions of different factors to overall variance in predicted CTG length. An extended discussion of this theme is present in section Limitations.``

5. It is as interesting, if not more, that only 29% of the AS defects seen can be explained by the repeat length alone. This is a strong finding given the statistical significance of the R2 in the results. Yet the authors do not comment on the biological relevance. It suggests that other components of the RBP complex may provide equal contributions to the weight of the pathology. Another alternative is that a minor fluctuation in MAL via a therapy would not be sufficient but rather significant knockdown would be required to repair AS. In any case, there should be discussion around the limited predictive value of the MAL on AS defects.

We have now added a new subsection in Limitations, "Weak correlations of predicted and true CTG length":

Correlations described in the study are quite modest, with effective CTG length as predicted from DM1-APA and the true CTG length as measured using PCR giving us the highest correlation with r^2^=0.29.

We would like to group potential reasons for this into two categories of factors, which capture the most likely reasons for weak correlations:

1. Weak	correlation of CTG repeat length with progressive DM1 symptoms in	general (potentially including the extent of AS defects). This	highlights a shortcoming of our current understanding of molecular	pathology of DM1.
2. Study	design, and in particular experimental techniques used in the study	introducing large amounts of technical noise.

While the first factor is well known (Thornton 2014), and a deeper understanding of DM1 pathomechanism, *e.g*. through further study of the RBPs is warranted, the study design (including experimental factors) is likely to be a major contributing factor.

Unfortunately, the study design did not include any biological or technical replicates, so we were unable to quantify the relative contributions of different factors to the error terms present in both measurements, and especially the measurements of AS defects. Consequently, we can only discuss the following qualitative reasons:

1. We are studying a complex, noisy system: atrophied muscle of individuals who volunteered in the study. These individuals exhibited difference in age (which would contribute to somatic mosaicism discrepancy between blood/muscle), sex, and would have different lifestyles. All these factors can contribute to DM1 pathomechanism in a way, which couldn't be captured with CTG repeat sizing in blood.

2. The platform we are using (Human Exon array) cannot detect all available signal (*e.g*. some of the short exons are not targeted). There are several other issues with the experimental setup: measurements are based on just 9 pixels from the original image from the scanner. Most probes have no technical replicates. The array used was not a junction array, it's difficult to attribute within-transcript change to any particular AS events/defects. Probe GC content can be a confounder (but the method we've used is resistant to any additive bias from GC content). Dyes used in microarrays are very sensitive to daily conditions in the laboratory, *e.g*. it has been shown that levels of atmospheric ozone has significant effect on the measurements.

3. The original list of biomarkers was discovered in different muscle groups.

4. Our model is quite simple -- it doesn't encode biological intuition behind alternative splicing or alternative polyadenylation. All features are treated as independent, even within a single transcript.

5. The repeat was sized in blood as opposed to muscle. Repeat sizing in muscle is very challenging, but would likely give stronger levels of signal.

6. An alternative approach not discussed is the measurement of MAL in muscle as likely having higher correlations (though technically more difficult).

This is now discussed in Limitations.

# Response to comments by Juan Antonio Carbonell-Asíns

1. Line #124: I would like to underline that the authors mention that the database has 35 patients but, nevertheless, line #127 the patients seem to be 36. It seems that the true sample was 36 but needs clarification as it is confusing.

Thank you for noticing this discrepancy. 36 participants were recruited in the study, but phlebotomy was successful for only 35 of these participants. One participant had to be thus excluded from the study as their repeat could not be sized. We have now corrected this discrepancy in the manuscript:

As part of the *Dystrophia Myotonica* Biomarker Discovery Initiative (DMBDI) microarray analysis was used to investigate mRNA profiles in quadriceps muscle from 36 participants, including 32 DM1 cases and four unaffected controls. One DM1 patient refused blood donation, thus genetic analyses were only performed on 35 participants. All DM1 cases genotyped were heterozygous for the expanded CTG repeat and the mode of the length of the DM1 CTG expansion (Modal Allele Length, MAL) was determined by small-pool PCR of blood DNA for 35/36 patients. For this work we did not attempt to measure the repeat length from muscle, due to a very high degree of repeat instability in muscle cells and associated difficulties in its experimental measurement.

2. Data preparation needs to be clearer. The authors could clarify which are the dependent and independent variables, as it is not easy to be deducted in the text. Pipeline description is not clear, i.e. what information provides the fact that there are 19,826 files? (#186-#212).

We have now expanded the Data preparation and analysis section.

3. Splitting data with only 28/35 patients may cause bias in estimations. (Xu, Y., & Goodacre, R., 2018). So, I would like to recommend using all patients and conduct a standard model diagnostic. No model diagnosis has been provided.

Model diagnostics could not have been productively used with our model of choice (feature selection followed by PLSR), due to the complexity of the model.

Using all data for training followed by model diagnostics would prevent us from obtaining any information about the predictive power of the model employed, and we decided it was ill-suited to the analytical task at hand.

We would like to stress that simple linear regression as visualised in Figure 1 **is not** a visualisation of the predictive model. It is a visualisation of the quality of predictions obtained from the model (across multiple repetitions of CV), and has little connection with the actual model delivering predictions (incidentally, also based on linear regression). We understand how the potential confusion might have arisen; we have expanded our "Data preparation and analysis" section to prevent this potential confusion from recurring.

We would like to stress that our selection of cross-validation strategy could not have resulted in a significant source of bias in predictions of CTG length. Even the reference cited, although focusing on a different analytical task (classification), specifically notes correctness of predictions (classifications), regardless of the parameters of the CV split:

"The results suggested that most methods with typical parameter settings resulted in similar correct classification results (Figs. 4, 5, 6, 7, 8, 9 and see ESM), therefore, they are all viable options for model selection."

We agree with the reviewer, that our choice of CV strategy could have resulted in underestimation of predictor's performance (as measured using r^2^). However, we do not see this as a major limitation of the study. Underestimating predictive power of the model allows us to draw a conservative conclusion with regards to the thesis we put forward in the study, which is the applicability of the measurement of AS biomarkers (in muscle biopsies), CTG repeat length sizing (in blood), and the proposed statistical framework in the context of clinical trials for emerging DM1 treatments.

4. I would like to suggest the authors to characterize the descriptive analysis provided, including but not limited to MAL variable.

Please see an improved description of the analysis carried out in the "Data preparation and analysis section".

5. R2 is used to evaluate predictive power of each model but this strategy does not penalize by number of predictors. Other methods are suggested as, for instance, AIC/BIC.

The R^2^ was computed between the predicted MAL (from the measurement of AS defects, using either PLSR or other models) and the true MAL (measured experimentally with repeat sizing). For the purpose of this computation there is only one "predictor" and only one "response". There is no need to penalise for the number of predictors.

While predicted MAL does come from several complex models, with variable number of predictors (up to 500), only predictions on the training set are used in the evaluation of the model. In this context penalising for the number of predictors is also unnecessary.

We've taken extreme caution to avoid double-dipping, i.e. the true value of MAL of data points in the testing fold is always hidden from the model during training, including at the feature selection stage.

6. I think that some clarification could be useful in understand how linear regression was conducted as it seems that there are almost the same number of predictors than patients for DM1-AS. Thus, R2 should be much higher than reported or even not calculable.

Please see an improved description of the analysis carried out in the "Data preparation and analysis" section

7. Relation between previous sections and power analysis section needs to be clearer.

We have now expanded the power subsection in the "Data preparation and analysis" section.
